# The *Allium* Derivate Propyl Propane Thiosulfinate Exerts Anti-Obesogenic Effects in a Murine Model of Diet-Induced Obesity

**DOI:** 10.3390/nu14030440

**Published:** 2022-01-19

**Authors:** Rebeca Liébana-García, Marta Olivares, Sonia M. Rodríguez-Ruano, Verónica Tolosa-Enguís, Isabel Chulia, Lidia Gil-Martínez, Enrique Guillamón, Alberto Baños, Yolanda Sanz

**Affiliations:** 1 Institute of Agrochemistry and Food Technology, Spanish National Research Council (IATA-CSIC), 46980 Valencia, Spain; reliegar@iata.csic.es (R.L.-G.); veronica.tolosa@iata.csic.es (V.T.-E.); ichumi@alumni.uv.es (I.C.); yolsanz@iata.csic.es (Y.S.); 2 Department of Microbiology, Faculty of Sciences, University of Granada, 18011 Granada, Spain; soniamrr@ugr.es; 3 DMC Research Center, 18620 Granada, Spain; lidiagm@dmcrc.com (L.G.-M.); enrique.guillamon@dmcrc.com (E.G.); abarjona@dmcrc.com (A.B.)

**Keywords:** obesity, microbiota, propyl propane thiosulfinate, *Allium*, dose effect

## Abstract

*Allium* species and their organosulfur-derived compounds could prevent obesity and metabolic dysfunction, as they exhibit immunomodulatory and antimicrobial properties. Here, we report the anti-obesogenic potential and dose-dependent effects (0.1 or 1 mg/kg/day) of propyl propane thiosulfinate (PTS) in a murine model of diet-induced obesity. The obesogenic diet increased body weight gain and adipocyte size, and boosted inflammatory marker (*Cd11c*) expression in the adipose tissue. Conversely, PTS prevented these effects in a dose-dependent manner. Moreover, the higher dose of PTS improved glucose and hepatic homeostasis, modulated lipid metabolism, and raised markers of the thermogenic capacity of brown adipose tissue. In the colon, the obesogenic diet reduced IL-22 levels and increased gut barrier function markers (*Cldn3, Muc2, Reg3g, DefaA*); however, the highest PTS dose normalized all of these markers to the levels of mice fed a standard diet. Gut microbiota analyses revealed no differences in diversity indexes and only minor taxonomic changes, such as an increase in butyrate producers, *Intestimonas* and *Alistipes*, and a decrease in *Bifidobacterium* in mice receiving the highest PTS dose. In summary, our study provides preclinical evidence for the protective effects of PTS against obesity, which if confirmed in humans, might provide a novel plant-based dietary product to counteract this condition.

## 1. Introduction

Obesity is characterized by excessive body fat accumulation, which increases the risk of suffering from chronic diseases such as heart failure, cancer, and type 2 diabetes [1]. Many single-nucleotide polymorphisms are associated with the development of obesity; however, these differences in the genetic background alone do not explain the incidence of obesity [2,3]. Indeed, the constant rise in obesity prevalence can only be explained by broad environmental changes, given the stasis of human biology within the same time frame [4]. Changes in food systems and dietary patterns undoubtedly influenced the epidemiology of obesity. In this respect, hypercaloric diets disrupt the energy balance, immune system, and gut microbiome, whereas a balanced, plant-based diet protects from fat deposition and metabolic impairment [5].

Some of the health benefits associated with the intake of vegetables are due to their content in bioactive compounds. Accordingly, the administration of extracts rich in certain bioactive compounds to obese human subjects [6,7,8] or rodents [9,10] is proven to reduce some of the features associated with obesity and its comorbidities (e.g., decreases in body weight and plasma cholesterol levels or improvements in waist/hip and LDL/HDL ratios). Among the bioactive compounds currently stirring interest are the organosulfur compounds obtained from vegetables of the genus *Allium*—such as onions, garlic, or leeks—synthesized by these plants as a defense mechanism against tissue damage. The fact that these organosulfur compounds have functional properties exerting antibacterial, antifungal, anti-inflammatory, or antioxidant activities might underlie the century-old folk wisdom that vouches for their beneficial properties [11,12].

The most widely studied organosulfur compound is the so-called allicin (diallyl thiosulfinate). In the context of obesity, allicin is shown to curb obesity in animal models, an effect that could be, at least partly, mediated by the gut microbiota [13,14]. However, one limitation of allicin is that it is precariously unstable, and its bioavailability raises doubts [15]. To a lesser extent, other organosulfur compounds from the *Allium* genus were also investigated, such as propyl-propane thiosulfonate (PTSO) or propyl propane thiosulfinate (PTS). In this respect, the compound PTSO displayed beneficial effects on two murine models of colitis—induced by the dextran sodium sulfate (DSS) and the 2,4-dinitrobenzene sulfonic acid (DNBS)—by reducing different pro-inflammatory mediators and improving the integrity of the intestinal epithelial barrier [16]. These two mechanisms are also pinpointed by Vezza et al. to explain the anti-obesogenic effects of this compound in a very recent in vivo study [17]. Regarding PTS, there is evidence of its potential use as an additive for animal nutrition [18] and its antimicrobial and anti-coccidial properties in vitro [19,20]. Moreover, PTS was demonstrated to be toxicologically safe in rats [21]. However, to our knowledge, research so far has not investigated PTS in the context of its potential protective effects on metabolic health.

In the present study, we describe the anti-obesogenic potential of the *Allium* derivate PTS, tested at two different doses in a murine model of diet-induced obesity. We describe how PTS administration prevents body weight gain and the metabolic impairment associated with a hypercaloric diet in a dose-dependent manner. We also shed light on its mode of action through the effects exerted on immune mediators and energy homeostasis.

## 2. Materials and Methods

### 2.1. Mice and Treatments

The experiments were performed with a total of 32 C57BL/6J mice (7-week-old males, Charles River Laboratories, Écully, France). Mice were housed in groups of four per individually ventilated cage under a 12 h light/dark cycle (lights on at 8:00) in a temperature-controlled room (23 ± 2 °C). Mice were acclimatized for 10 days with a standard diet. During the experiment, mice had ad libitum access to water and food. The experiment was approved and performed following European Union 2010/63/UE and Spanish RD53/201 guidelines, approved by the ethics committee of the University of Valencia (Animal Production Section, SCSIE, University of Valencia) and authorized by the competent authority (Generalitat Valenciana). Furthermore, all efforts were made to guarantee the three Rs before performing the experiment. Accordingly, we considered alternatives to animal experimentation (replacement), but given its unavoidability we paid attention to reducing the number (reduction) and minimizing the suffering of, or harmful effects (refinement) on, the experimental animals. The Generalitat Valenciana authorized the procedure under the title “*Evaluation of bioactive compounds obtained from plant sources*” and assigned the code 2020/VSC/PEA/0088 on 26 May 2020.

Randomization of mice into four experimental groups (*n* = 8) was conducted based on body weight to minimize baseline differences. For 10 weeks, mice were treated with: (1) control diet (D12450K Ssniff; 10% of energy from fat and no sucrose), (2) high-fat high-sugar diet (HFHSD, D12451 Ssniff; 45% of energy from lard, and 35% from sucrose), (3) HFHSD plus low-dose PTS (0.1 mg/kg/day), and (4) HFHSD plus high-dose PTS (1 mg/kg/day). Table S1 shows the detailed composition of the diets. PTS from *Allium cepa* L. (95% purity) was developed and provided by DOMCA SAU (Granada, Spain). The compound was administrated daily by oral gavage, whereas the control groups received PBS. Trends in body weight and food and water intake were monitored twice per week.

In the 10th week, animals were fasted, anesthetized with isoflurane, and sacrificed by cervical dislocation. Blood samples were collected in EDTA tubes, and plasma was immediately transferred after centrifugation (12,000× *g*, 3 min). One aliquot of plasma was kept on ice to assess intestinal permeability, and one aliquot was stored at −80 °C for biochemical and immune analyses. A section of white adipose tissue (WAT) was immersed in a solution of 4%paraformaldehyde for histological analysis. Liver, brown adipose tissue (BAT), WAT (subcutaneous (inguinal) and visceral (epididymal)), two sections of the intestine (ileum and colon), and the cecal content were snap-frozen in liquid nitrogen and stored at −80 °C until use.

### 2.2. Oral Glucose Tolerance Test

An oral glucose tolerance test (OGTT) was conducted at week 9 after 4 h fasting. Blood from the saphenous vein was collected at 0, 15, 30, 60, and 120 min after an oral glucose challenge (2 g/Kg). Glucose was measured using glucose test strips (CONTOUR^®^-Next meter, Bayer, Leverkusen, Germany).

### 2.3. Intestinal Permeability

Fluorescein isothicyanine (FITC)-dextran 4 kDa (Sigma-Aldrich, St. Louis, MO, USA, 600 mg/Kg) was administrated by oral gavage one hour before the sacrifice. Plasma was diluted in an equal volume of PBS, and fluorescence was measured at the excitation wavelength of 485 nm and 535 nm emissions (CLARIOstar^®^ Plus Multi-Mode Microplate Reader, Ortenberg, Germany). Standard curves were obtained by diluting FITC-dextran in the plasma of non-treated mice, as previously described [22].

### 2.4. Biochemical Analyses

Plasma levels of insulin, glucose, triglycerides, cholesterol, and non-esterified fatty acid (NEFA) were measured with the following commercial kits: ultrasensitive insulin ELISA kit (Mercodia, Uppsala, Sweden), glucose liquid (Química Analítica Aplicada SA, Tarragona, Spain), triglyceride colorimetric assay kit (Elabscience, Houston, TX, USA), cholesterol liquid kit (Química Analítica Aplicada SA, Spain), and NEFA colorimetric assay kit (Elabscience, USA), respectively. The HOMA-IR index was calculated as fasting plasma insulin (mU/L) × fasting plasma glucose (mmol/L)/22.5.

The citrate synthase activity was measured in the BAT using a colorimetric assay kit (BioVision, Milpitas, CA, USA). Hepatic glycogen was quantified according to the manufacturer’s instruction of a commercial kit (Sigma-Aldrich, USA). In addition, the lipid content was quantified in the liver after extraction with chloroform, as previously described [23]. Briefly, the tissues were homogenized in chloroform/methanol (2:1) solution. After 3 h of shaking, Milli-Q water was added, and the organic layer was separated by centrifugation (16,000× *g*, 20 min) and dried overnight. Triglyceride concentration was measured as described above.

### 2.5. Immune Parameters

One section of the colon and one section of the liver were homogenized in RIPA buffer (Sigma-Aldrich) with a protease inhibitor cocktail (Sigma-Aldrich). The homogenate was centrifuged (12,000× *g*, 10 min). In the supernatant from the colon and the liver, the levels IL-22 or IL-6 were, respectively, quantified with an ELISA kit (ELISA MAX^TM^) Deluxe Set, Biolegend, San Diego, CA, USA). The values were normalized with the amount of protein quantified using the Bradford method. Plasma levels of IL-6 were measured using the Luminex™ IL-6 Mouse Bead kit (Invitrogen, Waltham, MA, USA). In suspensions of the cecal content, secretory immunoglobulin A (sIgA) was measured using another commercial kit (Invitrogen, Waltham, MA, USA).

### 2.6. Gene Expression Analyses

Total RNA was isolated from different sections of the intestine (ileum and colon) using the commercial kit Nucleo Spin RNA (Macherey-Nagel, Nordrhein-Westfalen, Germany) and from the WAT and liver using the TRIsureTM reagent (Bioline, London, UK). Complementary DNA was prepared by the reverse transcription of 1 µg of total RNA using the kit High-Capacity cDNA Reverse Transcription (Applied Biosystems, Foster City, CA, USA), following manufacturer´s instructions. The RT-qPCR was performed with the LightCycler^®^ 480 Instrument (Roche, Boulogne-Billancourt, France). The reaction consisted of LightCycler 480 SYBR Green I Master mix (Roche) and 300 nM of gene-specific primer pairs. Samples were run in duplicate, and the data were analyzed using the 2^−ΔΔCT^ method. The qPCR program was previously described [23]. Targeted genes were normalized with the expression of ribosomal protein L19 (*Rpl19*), the housekeeping gene. Primer sequences are detailed in Table S2.

### 2.7. Histological Analysis

The visceral WAT was stained with hematoxylin/eosin to quantify the size of the adipocytes. The bright-field digital images were taken using an E90I Nikon microscope (Nikon Corporation, Tokio, Japan) with an 8x objective, equipped with a digital camera (Nikon DS-5Mc). A combined analysis was carried using Fiji (ImageJ 1.49q Software, National Institutes of Health, Bethesda, MD, USA) and Nis Elements BR 3.2 software (Nikon Corporation, Japan). The cross-sectional area of each adipocyte was automatically recognized and calculated by the NIS-elements software. Artifacts were manually discarded. Three independent measurements from different sections were performed on each mouse. At least 350 adipocytes were quantified per measurement.

### 2.8. Gut Microbiota Composition

Genomic DNA was extracted from the cecal content using a QIAamp PowerFecal DNA Kit (Qiagen, Hilden, Germany), and gut microbiota composition was analyzed by sequencing the V3-V4 hypervariable region of the 16S rRNA gene in an Illumina platform (MiSeq). The complete bioinformatics analysis is described in Supplementary Materials.

### 2.9. Statistical Analyses

G*Power 3.1.9.2 was used to calculate sample size and GraphPad software (version 8, San Diego, CA, USA) for statistical analyses and plots. Shapiro–Wilk test was employed to assess data normality. For normally distributed data, differences were determined with one-way analysis of variance (ANOVA) or two-way ANOVA (as suitable) followed by *post hoc* Tukey’s multiple comparison tests. Welch’s correction was applied when variances were not equally distributed, followed by a Dunnett’s T3 multiple comparisons test. Non-normally distributed data were analyzed with the Kruskal–Wallis test followed by Dunn’s multiple comparisons test. For gut microbiota analyses, Benjamini–Hochberg method was used to adjust *p*-values and Permutational Multivariate Analysis of Variance (adonis) followed by a pairwise *post-hoc* test (pairwiseAdonis). The results were considered statistically significant at *p* < 0.05. For all analyses, the Grubbs test was used for outlier detection.

## 3. Results

### 3.1. PTS Curbed Obesity by Influencing the White and Brown Adipose Tissue Metabolism in a Dose-Dependent Manner

**Figure 1:**
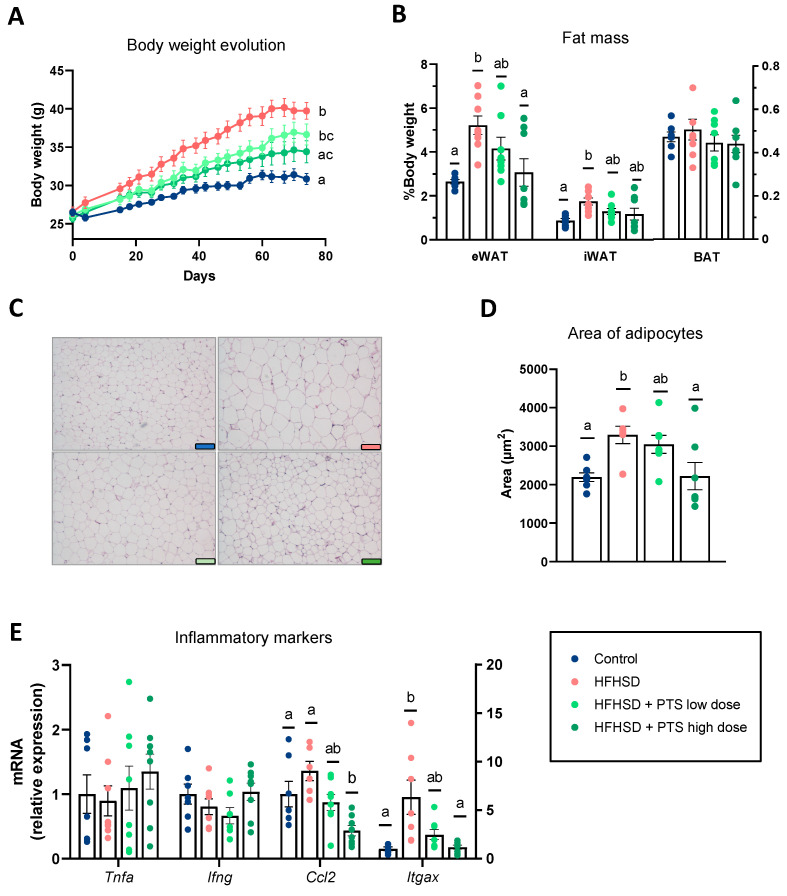
(**A**) Body weight development (two-way ANOVA *p* < 0.001), (**B**) weight of the fat mass: eWAT (Kruskal–Wallis *p* < 0.01) and iWAT (one-way ANOVA *p* < 0.05), (**C**) histology of the visceral adipose tissue, (**D**) area of the adipocytes (one-way ANOVA *p* < 0.01), and (**E**) expression of markers of inflammation: *Itgax* (Kruskal–Wallis *p* < 0.01) and *Ccl2* (Welch’s ANOVA *p* < 0.01). Mice were fed a control diet (*n* = 6–7); a high-fat, high-sucrose diet (HFHSD) (*n* = 6–8); and HFHSD with propyl propane thiosulfinate (PTS) at a low dose (0.1 mg/kg day) (*n* = 7–8) and a high dose (1 mg/kg day) (*n* = 7–8). Statistical analyses were performed by one-way analysis of variance (ANOVA) followed by *post hoc* Tukey’s multiple comparison tests for normally distributed data, except for body weight development, which was analyzed with a two-way ANOVA followed by Tukey’s *post hoc* test. Welch’s correction was applied when variances were not equally distributed. Non-normally distributed data were analyzed with the Kruskal–Wallis test followed by Dunn’s multiple comparisons test. Different superscript letters show statistical differences in the *post hoc* test when *p* < 0.05. *Ccl2* gene codes for MCP-1; the *Itgax* gene codes for CD11c.

The HFHSD significantly increased body weight (Figure 1A) due to the expansion of the epididymal and inguinal WAT (Figure 1B). The administration of PTS curbed obesity in a dose-dependent manner; however, this effect only reached the statistical cut-off point at the higher dose. Accordingly, the histological analysis of the white fat pads showed that, at this dose, PTS prevented the increase in the adipocyte size induced by the HFHSD (Figure 1C,D). The morphological changes caused by the obesogenic diet were accompanied by clear increases in the expression of *Itgax*, coding for CD11c, a marker of M1 macrophages that are infiltrated in the adipose tissue in obesity [24] (Figure 1E). Only PTS at the higher dose reduced the expression of *Itgax* and *Ccl2,* coding for the chemokine MCP-1, which mediates the recruitment of macrophages.

**Figure 2:**
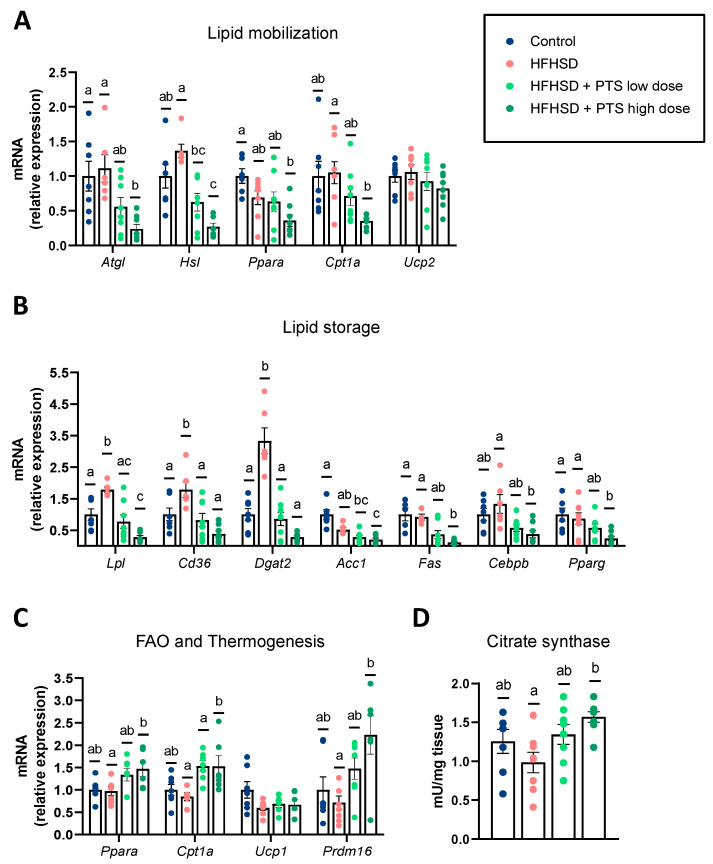
In the white adipose tissue: *Atgl* (one-way ANOVA *p* < 0.01)*, Hsl* (Kruskal–Wallis *p* < 0.001)*, Ppara* (one-way ANOVA *p* < 0.01) and *Cpt1a* (Welch’s ANOVA *p* < 0.01); (**A**) expression of markers of lipid mobilization: *Lpl* (Welch’s ANOVA *p* < 0.0001)*, Cd36* (one-way ANOVA *p* < 0.001)*, Dgat2* (Welch’s ANOVA *p* < 0.0001)*, Acc1* (Kruskal–Wallis *p* < 0.001)*, Fas* (Kruskal–Wallis *p* < 0.001)*, Cebpb* (Kruskal–Wallis *p* < 0.01) and *Pparg* (Kruskal–Wallis *p* < 0.01); and (**B**) lipid storage. In the brown adipose tissue, (**C**) expression of fatty acid oxidation (FAO) and thermogenesis: *Ppara* (one-way ANOVA *p* < 0.05)*, Cpt1a* (one-way ANOVA *p* < 0.01) and *Prdm16* (Kruskal–Wallis *p* < 0.05); and (**D**) citrate synthase activity (one-way ANOVA *p* < 0.05). Mice were fed a control diet (*n* = 6–7); a high-fat, high-sucrose diet (HFHSD) (*n* = 6–8); and HFHSD with propyl propane thiosulfinate (PTS) at a low dose (0.1 mg/kg day) (*n* = 6–8) and a high dose (1 mg/kg day) (*n* = 6–8). Statistical analyses were performed by one-way analysis of variance (ANOVA) followed by *post hoc* Tukey’s multiple comparison tests for normally distributed data, and Welch’s correction was applied when variances were not equally distributed. Non-normally distributed data were analyzed with the Kruskal–Wallis test followed by Dunn’s multiple comparisons test. Different superscript letters show statistical differences in the *post hoc* test when *p* < 0.05.

We also analyzed the expression of molecular markers involved in fat mobilization (Figure 2A) and storage (Figure 2B). In the WAT, the HFHSD did not modify fat mobilization, but it significantly upregulated pathways related to fat uptake and storage, such as *Lpl*, *Cd36*, and *Dgat2*. In contrast, the effect of PTS on lipid metabolism was balanced, with reductions in either fat mobilization and storage (Figure 2A,B). Specifically, the higher dose of PTS reduced the expression of markers involved in catabolic processes such as lipolysis (*Atgl*, *Hsl*), and beta-oxidation (*Cpt1a*); and in anabolic processes such as fatty acid uptake (*Lpl, Cd36),* fatty acid synthesis (*Acc1*, *Fas*), and adipogenesis (*Cebpb*, *Pparg*). However, PTS did not counteract the decline in markers that were caused by the obesogenic diet (Supplementary Figure S1). All the changes attributed to PTS administration occurred without any differences in the water or food intake, or in the plasmatic levels of triglycerides, NEFA, or cholesterol (Table S3).

Finally, we investigated whether the anti-obesogenic effect of PTS could be attributed to a higher thermogenic activity of the BAT. In agreement with our hypothesis, PTS increased the expression *Cpt1a* (the low and high dose) as well as *Ppara* and *Prdm16* (at the higher dose) (Figure 2C). Hence, to confirm the capacity of PTS to increase the thermogenic activity of the BAT, we analyzed the mitochondrial activity by quantifying the citrate synthase activity. Accordingly, we found that PTS boosted this activity in a dose-dependent manner, which was only statistically different between the group treated with the highest dose of PTS versus the obese group (Figure 2D).

### 3.2. PTS Improved Systemic Glucose Homeostasis, Hepatic Metabolism and Inflammatory Response

**Figure 3:**
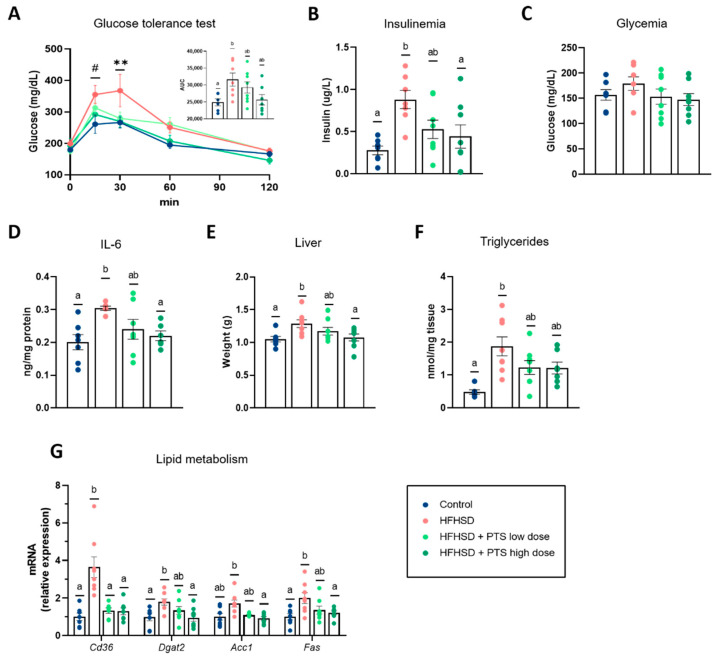
Systemic circulation: (**A**) Glucose tolerance test (two-way ANOVA *p* < 0.001); AUC (one-way ANOVA *p* < 0.05), *(***B**) Insulinemia (one-way ANOVA *p* < 0.01), and (**C**) glycemia. In the liver: (**D**) hepatic IL6 levels (Welch’s ANOVA *p* < 0.001)*,* (**E**) weight of the liver (one-way ANOVA *p* < 0.05), (**F**) triglycerides (Kruskal–Wallis *p* < 0.01), and (**G**) expression of markers of lipid metabolism: *Cd36* (Welch’s ANOVA *p* < 0.01)*, Dgat2* (one-way ANOVA *p* < 0.05)*, Acc1* (one-way ANOVA *p* < 0.01), and *Fas* (one-way ANOVA *p* < 0.05). Mice were fed a control diet (*n* = 6–7); a high-fat, high-sucrose diet (HFHSD) (*n* = 6–8); and HFHSD with propyl propane thiosulfinate (PTS) at a low dose (0.1 mg/kg day) (*n* = 7–8) and at a high dose (1 mg/kg day) (*n* = 7–8). Statistical analyses were performed by one-way analysis of variance (ANOVA), followed by *post hoc* Tukey’s multiple comparison tests for normally distributed data, and Welch’s correction was applied when variances were not equally distributed. Non-normally distributed data were analyzed with the Kruskal–Wallis test, followed by Dunn’s multiple comparisons test. Different superscript letters show statistical differences in the *post hoc* test when *p* < 0.05. “*” means significantly different within all groups, “#” means significantly different compared with the control group. ** *p* < 0.01; and ^#^ *p* < 0.05.

High-calorie diets are well-known to impair glucose metabolism. Here, we confirmed that mice fed an HFHSD had a worse response to the glucose tolerance test and exhibited uncontrolled insulinemia (Figure 3A,B). The administration of PTS tended to attenuate both effects in a dose-dependent manner. Moreover, even if fasting glycaemia and the hepatic glycogen levels remained unchanged between groups (Figure 3C and Figure S2A), the metabolic disruption in response to the HFHSD led to a pre-diabetic state, as estimated by the HOMA-IR index, which was not observed in PTS-treated animals (Figure S2B).

Moreover, the HFHSD triggered hepatic steatosis, as evidenced by an increase in the inflammatory tone (assessed by IL-6 quantification) (Figure 3D) and higher hepatic weight due to triglycerides accumulation (Figure 3E,F). No changes were detected in the plasmatic levels of IL-6 (Figure S2C). Meanwhile PTS at the highest dose significantly prevented both effects. Furthermore, the molecular analysis of the liver showed that the HFHSD upregulated lipid storage markers (*Cd36*, *Dgat2*, and *Fas*), which all remained at the same level as the control group in mice treated with the highest dose of PTS (Figure 3G).

### 3.3. PTS Had Minor Effects on the Intestinal Permeability and the Gut Barrier Function

**Figure 4:**
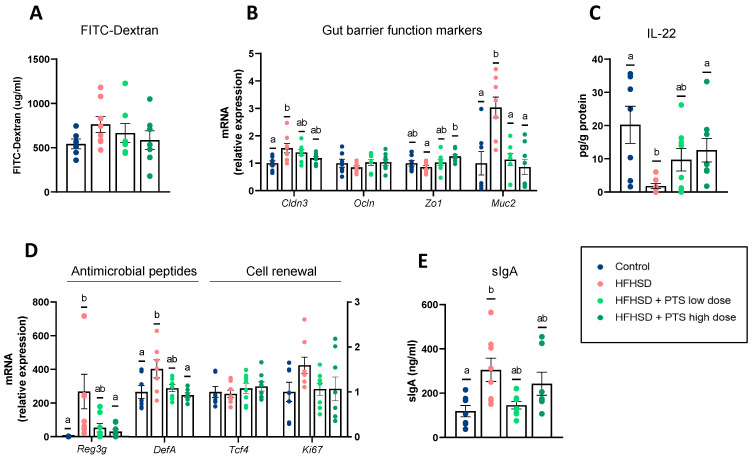
Gut barrier function assessed (**A**) by FITC translocation in the systemic circulation, and (**B**) expression of tight junction markers and mucin in the colon: *Cldn3* (one-way ANOVA *p* < 0.05)*, Zo1* (one-way ANOVA *p* < 0.05) and *Muc2* (one-way ANOVA *p* < 0.0001), (**C**) levels of IL-22 in the colon (Kruskal–Wallis *p* < 0.01), and (**D**) expression of antimicrobial peptides and cell renewal markers: *Reg3g* (Kruskal–Wallis *p* < 0.01) and *DefA* (one-way ANOVA *p* < 0.05). In the cecal content, (**E**) levels of secretory immunoglobulin A (sIgA) (Kruskal–Wallis *p* < 0.05). Mice were fed a control diet (*n* = 6–7); a high-fat, high-sucrose diet (HFHSD) (*n* = 7–8); and HFHSD with propyl propane thiosulfinate (PTS) at a low dose (0.1 mg/kg day) (*n* = 7–8) and at a high dose (1 mg/kg day) (*n* = 7–8). Statistical analyses were performed by one-way analysis of variance (ANOVA) followed by *post hoc* Tukey’s multiple comparison tests. Non-normally distributed data were analyzed with the Kruskal–Wallis test followed by Dunn’s multiple comparisons test. Different superscript letters show statistical differences in the *post hoc* test when *p* < 0.05.

The in vivo assessment of intestinal permeability showed no differences caused either by diet or by PTS (Figure 4A). The HFHSD significantly altered the expression of genes involved in barrier function in the colon (*Cldn3*, *Zo1,* and *Muc2*) (Figure 4B), and ileum (*Cldn3* and *Ocln*) (Figure S3A). In contrast, PTS at the highest dose restored the expression of *Zo1* and *Muc2* to the level of the control group (Figure 4B). Based on the role attributed to IL-22 in the gut barrier function [25], we measured this cytokine in the colon. The group fed an HFHSD presented a sharp drop in IL-22 levels, which the highest dose of PTS completely counteracted (Figure 4C). Additionally, in the colon, the HFHSD impaired the expression of the antimicrobial peptides (AMPs), known as *Reg3g* and *DefA*, both restored by the highest dose of PTS (Figure 4D). There were no changes in the expression of cell renewal markers in the colon (Figure 4D) or of AMPs in the ileum (Figure S3B). Lastly, in terms of the cecal content, we quantified the sIgA and observed an increase linked to the obesogenic diet, which was not prevented by PTS at either of the doses tested (Figure 4E).

### 3.4. PTS Caused No Differences in Gut Microbiota Diversity and Only Minor Taxonomic Changes

**Figure 5:**
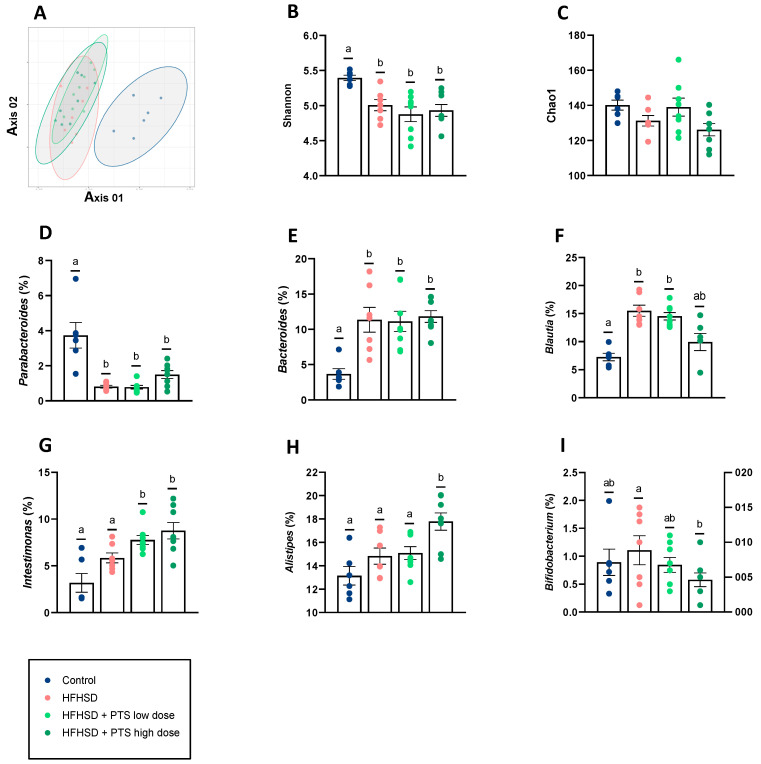
(**A**) NMDS showing the beta-diversity based on Bray–Curtis dissimilarity, (**B**) Shannon (Kruskal–Wallis *p* < 0.01) and (**C**) Chao1 diversity indexes, genera modified by the obesogenic diet. (**D**) *Parabacteroides* (control vs HFHSD q < 0.001; control vs HFHSD + PTS low dose q < 0.001; control vs HFHSD + PTS high dose q < 0.01), (**E**) *Bacteroides* (control vs HFHSD q < 0.001; control vs HFHSD + PTS low dose q < 0.001; control vs HFHSD + PTS high dose q < 0.001), (**F**) *Blautia* (control vs HFHSD q < 0.001; control vs HFHSD + PTS low dose q < 0.001), genera modified by the compound propyl propane thiosulfinate (PTS), (**G**) *Intestimonas* (control vs HFHSD + PTS low dose q < 0.001; control vs HFHSD + PTS high dose q < 0.001; HFHSD vs HFHSD + PTS low dose q < 0.05; HFHSD vs HFHSD + PTS high dose q < 0.05), (**H**) *Alistipes* (control vs HFHSD + PTS high dose q < 0.001; HFHSD vs HFHSD + PTS high dose q < 0.05, HFHSD + PTS low dose vs HFHSD + PTS high dose q < 0.05), (**I**) *Bifidobacterium* (HFHSD vs HFHSD + PTS high dose q < 0.001). Mice were fed a control diet (*n* = 6–7); a high-fat, high-sucrose diet (HFHSD) (*n* = 7–8); HFHSD with PTS at low dose (0.1 mg/kg/day) (*n* = 8); and HFHSD + PTS high dose (1 mg/kg/day) (*n* = 8). Taxonomic composition differences were statistically tested by Analysis of Compositions of Microbiomes with Bias Correction (ANCOM-BC). Different superscript letters show statistical differences in the *post hoc* test when *p* < 0.05 or q < 0.05.

Non-metric multidimensional scaling (NMDS) showing the beta-diversity based on the Bray–Curtis dissimilarity indicated that the samples clustered based on the diet (control versus HFHSD) (Figure 5A). Accordingly, the three experimental groups that received the HFHSD presented a significant reduction in the Shannon index (Figure 5B) but no changes in the Chao1 diversity index (Figure 5C). When considering the changes in those OTUs identified at the genus level, the intake of HFHSD, independently of whether it was accompanied with the PTS treatment or not, caused decreases in *Parabacteroides* (Figure 5D) and increases in *Bacteroides* (Figure 5E) and *Blautia* (except at the high dose) (Figure 5F). Conversely, the administration of PTS at both doses was only associated with increases in the bacterial genus *Intestimonas* (Figure 5G). Additionally, the highest dose of PTS increased *Alistipes* (Figure 5H) and reduced the relative abundance of *Bifidobacterium* (Figure 5I).

## 4. Discussion

Plants belonging to the genus *Allium* were used in ancient medicine for the management of several health conditions. Nowadays, we have scientific evidence that supports this empirical knowledge. For instance, a recent meta-analysis including sixteen randomized clinical trials concluded that garlic supplementation reduces the level of circulating inflammatory markers (C-reactive protein, TNF, and IL-6) [26]. The perspective of obesity as an inflammatory condition justifies the search for compounds that counteract inflammation and their subsequent use as new therapeutic tools in this context [27]. Based on the anti-inflammatory properties recently attributed to some *Allium*-derived compounds [16,17], we evaluated the potential effects of PTS in an animal model of diet-induced obesity for the first time. Our results show that PTS prevented adiposity and improved several metabolic parameters through a network of interconnected mechanisms, including anti-inflammatory effects, thermogenesis induction, and gut homeostasis protection.

High-calorie diets lead to gradual body weight gain due to fat deposition. This expansion of adipose tissue is accompanied by adipose macrophage recruitment and inflammation, which are described in other studies and confirmed here [10,24,28]. Specifically, we describe the potential of PTS to reduce the expansion of the adipose tissue via the downregulation of inflammatory immune cells and chemokines (*Ccl2, Itgax*) and of genes involved in lipid storage (*Cd36*, *Lpl* and, *Dgat2*). The HFHSD also caused ectopic fat accumulation in the liver that, similarly to what is observed in the adipose tissue, can respond to the overexpression of genes involved in lipid storage and lipogenesis. Hepatic steatosis and pro-inflammatory responses, such as those mediated by IL-6, are frequently accompanied by impaired glucose homeostasis [29,30,31,32,33]. Accordingly, HFHSD-fed mice displayed an increase in fasting insulinemia and had a worse response to the glucose tolerance test. Conversely, the prevention of fatty liver, together with the down-regulation of pro-inflammatory mediators caused by PTS in the adipose tissue (*Ccl2, Itgax*) and liver (IL-6), might have buffered the dietary effects and protected metabolic health, as shown by the better response of PTS-fed mice in the OGTT. In line with our observations, another study reported similar results with the related compound PTSO, which reduced the expression of IL-6 in the liver and inhibited the infiltration of macrophages in the fat and liver [17]. As in our case, the authors of the aforementioned study attribute the anti-obesogenic effect of their compound to its action on these pathways.

Together with the prevention of inflammatory responses, PTS administration caused a sharp increase in the thermogenic activity of BAT. Similarly, the anti-obesogenic properties of other plant extracts, such as rhubarb or camu camu, were linked to the potential of promoting energy expenditure [10,34]. Moreover, and more specifically, in obese mice treated with allicin, Zhang et al. observed an increase in the thermogenesis of BAT and an inhibition of the decrease in body temperature after cold stimulation [13]. In humans, the activity of brown fat is negatively correlated to body mass index and positively correlated to glucose tolerance and insulin sensitivity [35]. For this reason, the induction of BAT activity is one of the strategies used to increase energy expenditure and combat obesity [36,37], and in the specific case of our study, might have contributed to the lower body weight gain displayed by the mice treated with PTS.

The third mechanism explaining the protective effects of the PTS compound reported herein is related to the preservation of intestinal homeostasis. Although we found no differences in the FITC-dextran test, which measures intestinal permeability, we did find changes in markers of intestinal immune homeostasis. The most notable change concerns the levels of IL-22. In line with our observations, in previous studies, a reduction in IL-22 was associated with hypercaloric diets [38]. Moreover, this cytokine was closely associated with the regulation of antimicrobial peptides [25]; however, in our study, the obesogenic diet caused an increase in the AMPs expression of *Reg3g*, and *DefA*. This paradoxical behavior can be explained by the fact that AMPs production does not depend solely on the cytokine IL-22 as demonstrated, for instance, by the absence of differences in the expression of AMPs between IL-22 KO and wild-type mice [39]. Our study suggests that PTS administration could reverse the changes caused by an obesogenic diet in both IL-22 and AMPs, as one of the most plausible mechanisms explaining its protective effect against obesity.

Gut microbiota changes were proposed to explain the beneficial effects of other organosulfur compounds from the genus *Allium* in the context of obesity. For instance, in the case of allicin, a study transferring feces from mice treated with allicin to new recipients showed that the preventive effects exerted by the organosulphur compound in the former were mimicked in the latter [13]. In the present study, PTS did not substantially change the microbial ecosystem, as concluded by the results of the diversity indexes and the limited taxonomical modifications, suggesting that its protective effects are not primarily mediated by the microbiota. One of the changes consisted of an increase in *Intestimonas,* a butyrate producer, due to PTS administration at the highest dose. The impact of this genus on metabolic health has received little attention, with one of the few studies describing reductions in obese subjects suffering from binge-eating disorders [40]. Our observation that *Bifidobacterium* was reduced by PTS seems to contrast with the findings of other authors, who describe increases in this genus with allicin [13] and PTSO [17] in animal models of obesity. However, an evaluation of the antimicrobial activity exerted by PTS in vitro showed its ability to affect the growth of bifidobacteria [41]. Thus, we can speculate that the reductions in the genus *Bifidobacterium* could be due to a direct and specific effect of PTS on this bacterial group, rather than being the consequence of other indirect mechanisms.

Recently, the genotoxicity of PTS in male and female rats was assessed and 55 mg/kg was established as the maximum safe dose [21]. In the present study, we evaluated two dosages (0.1 or 1 mg/kg), which were far below this maximum threshold, and were thus considered safe. Safety concerns aside, these two doses displayed differences in their potential to prevent metabolic alterations in response to an obesogenic diet in this pre-clinical trial.

## 5. Conclusions

Overall, our study provides pre-clinical evidence for the protective effects and mode of action of PTS in an animal model of diet-induced obesity in a dose-dependent manner. Specifically, we demonstrate that the administration of PTS at the highest dose evaluated can prevent body weight gain, adiposity, and alterations in glucose metabolism. The observed effects were mainly attributed to the potential of the compound to reduce inflammation in peripheral tissues, maintain hepatic and intestinal homeostasis and increase thermogenic activity in BAT. In the future, these protective effects of PTS against obesity should also be confirmed in humans.

## Data Availability

Amplicon sequences of the 16S rRNA gene of study microbiotas were uploaded to ENA database under project accession number PRJEB50178. Raw data of the parameters analyzed in the study have also been made available as Supplementary Materials.

## Supplementary Materials

The following supporting information can be downloaded at: https://www.mdpi.com/article/10.3390/nu14030440/s1, Supplementary material and methods. Table S1: Composition of the diets used in this study. Table S2: Primer sequences for gene expression analyses by RT-PCR. Table S3: Total food and water intakes and plasmatic parameters. Figure S1: Markers in the white adipose tissue. Figure S2: (A) hepatic levels of glycogen, (B) index of insulin resistance addressed by the HOMA-IR, (C) plasmatic levels of IL-6. Mice were fed a control diet. Figure S3: In the ileum, gene expression of (A) markers of gut barrier function and, (B) antimicrobial peptides. Excel files of the raw data of all assessments included in the manuscript and in supplementary material.
