# Supplementary material for: The Allium Derivate Propyl Propane Thiosulfinate Exerts Anti-Obesogenic Effects in a Murine Model of Diet-Induced Obesity
Source: Nutrients. 2022 Jan 19;14(3):440. doi: 10.3390/nu14030440 (PMC8839906; doi:10.3390/nu14030440)
Supplement: Supplementary file 1 [file nutrients-14-00440-s001.zip › nutrients-1540772-supplementary.pdf]

Supplementary material and methods

Gut microbiota composition

Samples were sequenced at IPBLN-CSIC in an Illumina MiSeq run (PE 2x300 output mode) using the 16S ProV3V4 primer pair (amplicon size about 600 bp) (1) and the Nextera XT v2 Index kit for multiplexing. The mean sequencing depth was 140.000 raw reads per sample. Demultiplexed reads were processed using USEARCH v9.2.64 (2). Briefly, R1 and R2 reads were merged (with the parameters minmergelen set to 400 and maxmergelen set to 460) and sequences were trimmed to an equal length of 424 bp after primer removal and prior to quality filtering (maxee parameter set to 2). Finally, chimeras were removed and de novo OTU picking performed (global alignment option set to 97% identity). Taxonomy was assigned using BLAST+ algorithm v.2.12 against the SILVA 132 database (3). Very low abundant OTUs (as recommended in (4)), and all non-bacterial, chloroplast, and mitochondrial OTUs were filtered out using QIIME 1.9 (5). Data was rarefied at 10000 sequences per sample. No samples were discarded up to this step, so a total of 31 samples were used in the subsequent analyses. Alpha-diversity was estimated by the Chao1 index (for OTU richness) and the Shannon index (for OTU abundance and evenness) as calculated in QIIME 1.9 (5). Statistical analyses and plots were performed in R (6) using the following packages: phyloseq (7), microbiome (8), vegan (9), FSA (10), pairwiseAdonis (11), ANCOMBC (12), and ggplot2 (13).

References:

1- Takahashi, S., Tomita, J., Nishioka, K., Hisada, T., and Nishijima, M. (2014). Development of a prokaryotic universal primer for simultaneous analysis of Bacteria and Archaea using Next-Generation Sequencing. *PLOS ONE* 9, e105592. doi:10.1371/journal.pone.0105592.

2- Edgar RC. UPARSE: highly accurate OTU sequences from microbial amplicon reads. Nat Methods. octubre de 2013;10(10):996-8.

3 Pruesse E, Quast C, Knittel K, Fuchs BM, Ludwig W, Peplies J, et al. SILVA: a comprehensive online resource for quality checked and aligned ribosomal RNA sequence data compatible with ARB. Nucleic Acids Res. 2007;35(21):7188-96.

4. Bokulich NA, Subramanian S, Faith JJ, Gevers D, Gordon JI, Knight R, et al. Quality-filtering vastly improves diversity estimates from Illumina amplicon sequencing. Nat Methods. 2013;10(1):57-9.

5. Caporaso JG, Kuczynski J, Stombaugh J, Bittinger K, Bushman FD, Costello EK, et al. QIIME allows analysis of high-throughput community sequencing data. Nat Methods;7(5):335-6.

6. R: a language and environment for statistical computing: https://www.gbif.org/es/tool/81287/r-a-language-and-environment-for-statistical-computing

7. McMurdie PJ, Holmes S. phyloseq: An R Package for Reproducible Interactive Analysis and Graphics of Microbiome Census Data. PLOS ONE. 2013;8(4):e61217.

8. Lahti, L., and Shetty, S. (2012-2019). *Microbiome R package*. Available at: http://microbiome.github.io/.

9. Oksanen J, Blanchet FG, Kindt R, Legendre P, Minchin P, O'Hara R, et al. vegan: Community Ecology Package. CRAN-The Comprehensive R Archive Network.

10. Simple Fisheries Stock Assessment Methods. http://derekogle.com/FSA/

11. ArbizuPM.pairwiseAdonis: https://github.com/pmartinezarbizu/pairwiseAdonis/blob/ece560d2364992e5751bd3143 1f505805e29f73d/pairwiseAdonis/R/pairwise.adonis.R
12. Lin, H., and Peddada, S. D. (2020). Analysis of compositions of microbiomes with bias correction. *Nat. Commun.* 11, 3514. doi:10.1038/s41467-020-17041-7.
13. Pedersen HW Danielle Navarro, and Thomas Lin. Welcome ggplot2: https://ggplot2-book.org/

Table S1: Composition of the diets used in this study

	Control diet (g/100g)	HSHFD (g/100g)
**Composition (%)**		
Casein	20.7	25
Lard	1.6	20.8
Soybean oil	2.4	2.8
Corn starch	48.85	7
Maltodextrin	14	11
Sucrose	-	20.17
Cellulose powder	5	5.7
L-cysteine	0.25	0.3
Vitamin mixture	1	1
Mineral and trace elements mixture	6	6
**Energy content (% kJ)**		
Fat	10	45
Protein	20	20
Carbohydrates	70	35

High-fat high-sucrose diet, HSHSD

Table S2: Primer sequences for gene expression analyses by RT-PCR

**Targeted gene**	**Sequence S (5′-3′)**
Cldn3	F: TCATCGGCAGCAGCATCATCAC<br>R: ACGATGGTGATCTTGGCCTTGG
Zo1	F: TTTTTGACAGGGGGAGTGG<br>R: TGCTGCAGAGGTCAAAGTTCAAG
Occludin	F: ATGTCCGGCCGATGCTCTC<br>R: TTTGGCTGCTCTTGGGTCTGTAT
Tnfa	F: TGTCTCAGCCTCTTCTCATTCC<br>R: TGAGGGTCTGGGCCATAGAAC
Reg3g	F: TTCCTGTCCTCCATGATCAAA<br>R: CATCCACCTCTGTTGGGTTC
DefA	F: GGTGATCATCAGACCCCAGCATCAGT<br>R: AAGAGACTAAAACTGAGGAGCAGC
Tcf4	F: ATGGCAAACAGAGGAACTGG<br>R: GCCTGCTGAGAGTGAAGGAG
Ki67	F: CAGACTTGCTCTGGCCTACC<br>R: GGTTGGCGTTTCTCCTCTTT
Ifng	F: TTCTTCAGCAACAGCAAGGC<br>R: ACTCCTTTTCCGCTTCCTGA
Ccl2	F: GCAGTTAACGCCCCACTCA<br>R: CCCAGCCTACTCATTGGGATCA
Itgax	FACGTCAGTACAAGGAGATGTTGGA<br>R: ATCCTATTGCAGAATGCTTCTTTACC
Cpt1a	F: TTTGAATCGGCTCCTAATGG<br>R: CCCAAGTATCCACAGGGTCA
Acc1	F: TAATGGGCTGCTTCTGTGACTC<br>R: CTCAATATCGCCATCAGTCTTG
Fas	F: GGAGGTGGTGATAGCCGGTAT<br>R: TGGGTAATCCATAGAGCCCAG
Cd36	F: GCCAAGCTATTGCGACATGA<br>R: ATCTCAATGTCCGAGACTTTTCAAC
Dgat2	F: GCGCTACTTCCGAGACTACTT<br>R: GGGCCTTATGCCAGGAAACT
Atgl	F: CAACGCCACTCACATCTACGG<br>R: GGACACCTCAATAATGTTGGCAC
Hsl	F: ATGCCACTCACCTCTGATCC<br>R: CTGTCCTGTCCTTCCCGTAG
Pla2ga	F: AAGGATCCCCCAAGGATGCCAC<br>R: CAGCCGTTTCTGACAGTTCTGG
Pparg	F: GCATGGTGCCTTCGCTGA<br>R: TGGCATCTCTGTGTCAACCATG
Prdm16	F: CAGCACGGTGAAGCCATTC<br>R: GCGTGCATCCGCTTGTG
Ucp1	F: ACTGCCACACCTCCAGTCATT<br>R: CTTTGCCTCACTCAGGATTGG
Ucp2	F: ACCTTTAGAGAAGCTTGACC<br>R: TTCTGATTTCCTGCTACCTC
Cidea	F: TGCTCTTCTGTATCGCCCAGT<br>R: GCCGTGTTAAGGAATCTGCTG
Ppara	F: GATGTCACACAATGCAATTC

	R: CAGTTTCCGAATCTTTCAGG
Pgc1a	F: AGCCGTGACCACTGACAACGAG<br>R: GCTGCATGGTTCTGAGTGCTAAG
Muc2	F: CCCAGAAGGGACTGTGTATG<br>R: TGCAGACACACTGCTCACA

Table S3 : Total food and water intakes and plasmatic parameters

	Control diet	HFHSD	HFHSD + PTS low dose	HFHSD + PTS high dose
Total food intake (g/mouse)	81.7 ± 7.4	138.9 ± 12.5	149.3 ± 16.9	135.2 ± 13.1
Total water intake (ml/mouse)	155.6 ± 11.3	163.1 ± 10.1	165.7 ± 8.9	169.1 ± 16.7
Triglycerides	75.6 ± 5.9	60.2 ± 6.4	55.1 ± 4.6	56.5 ± 6.7
Cholesterol	162.1 ± 5.4[a]	208.2 ± 14.5[b]	197.3 ± 12.3[ab]	190.5 ± 8.3[ab]
NEFA	81.3 ± 6.5[a]	67.1 ± 5.2[ab]	64.1 ± 4.6[ab]	59.0 ± 5.4[b]

Mean and standard error. Mice were fed a control diet, a high-fat high-sucrose diet (HFHSD), HFHSD with propyl propane thiosulfinate (PTS) at low dose (0.1 mg/kg/day) and high dose (1 mg/kg/day). Statistical analyses were performed by Kruskal-Wallis for non-normally distributed data and one-way analysis of variance (ANOVA) followed by post hoc Tukey's multiple comparison tests. Different superscript letters represent statistical differences. Non-esterified fatty acid, NEFA.

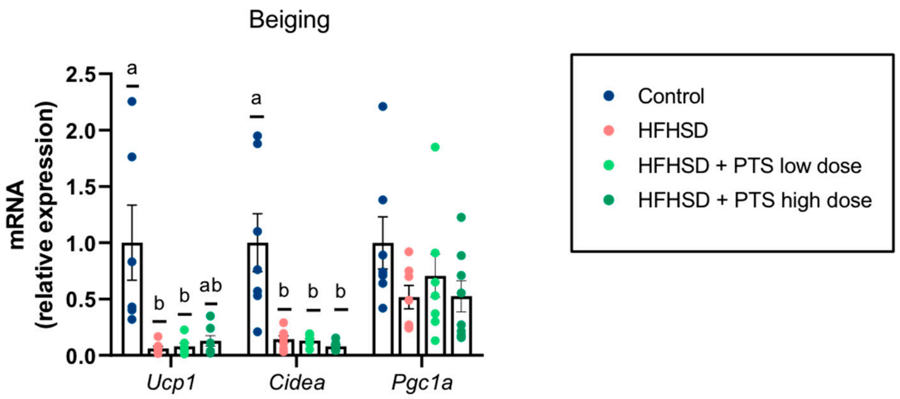

Figure S1: Markers of beiging in the white adipose tissue: *Ucp1* (Kruskal-Wallis p<0.01) and *Cidea* (Kruskal-Wallis p<0.001). Mice were fed a control diet (n=6-7), a high-fat high-sucrose diet (HFHSD) (n=7-8), HFHSD with propyl propane thiosulfinate (PTS) at low dose (0.1 mg/kg day) (n=7-8) and high dose (1 mg/kg day) (n=7-8). Statistical analyses were performed by one-way analysis of variance (ANOVA) followed by *post hoc* Tukey's multiple comparison tests. Non-normally distributed data were analyzed with the Kruskal-Wallis test followed by Dunn's multiple comparisons test Different superscript letters show statistical differences in the *post hoc* test when p<0.05.

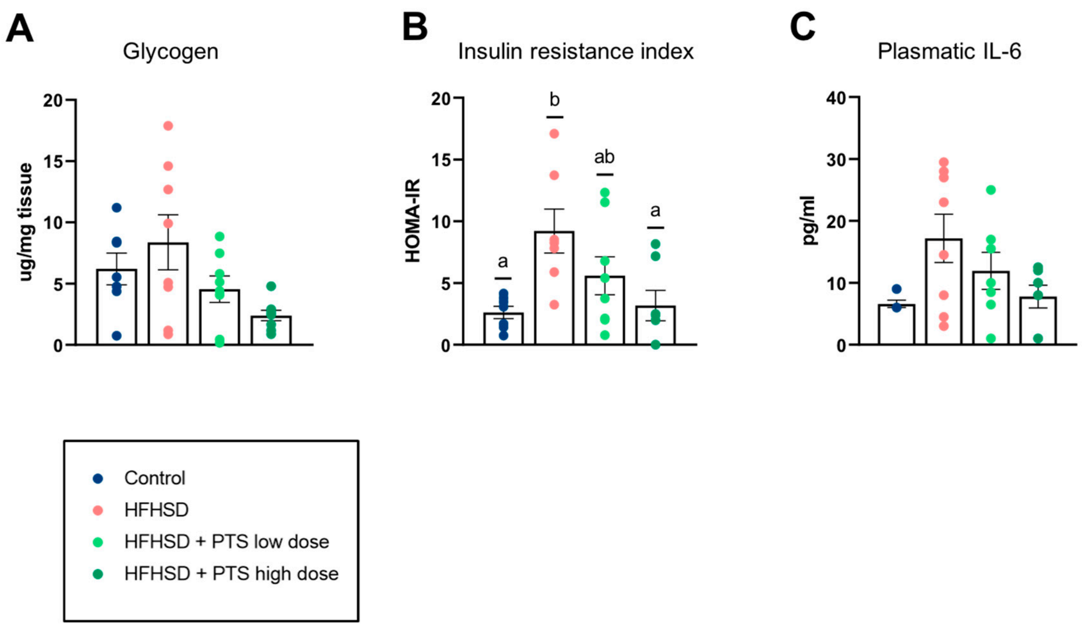

Figure S2. A) hepatic levels of glycogen, **B)** index of insulin resistance addressed by the HOMA-IR (one-way ANOVA $p<0.05$), **C)** plasmatic levels of IL-6. Mice were fed a control diet (n=5-7), a high-fat high-sucrose diet (HFHSD) (n=7-8), HFHSD with propyl propane thiosulfinate (PTS) at low dose (0.1 mg/kg day) (n=7-8) and high dose (1 mg/kg day) (n=7-8). Statistical analyses were performed by one-way analysis of variance (ANOVA) followed by *post hoc* Tukey’s multiple comparison tests. Non-normally distributed data were analyzed with the Kruskal-Wallis test followed by Dunn's multiple comparisons test Different superscript letters show statistical differences in the *post hoc* test when $p<0.05$.

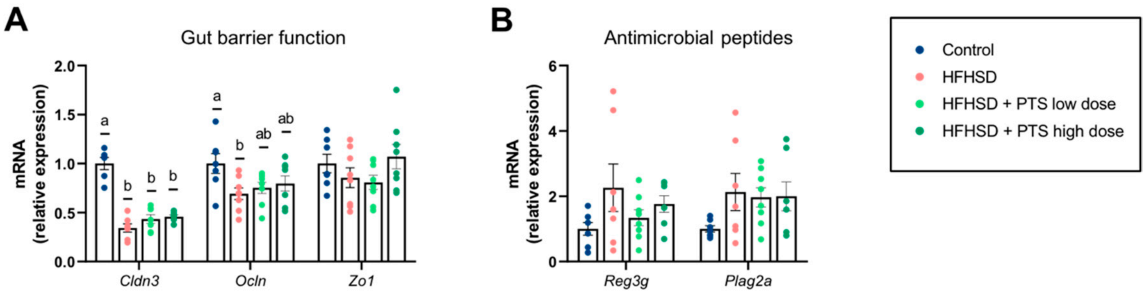

Figure S3: In the ileum, gene expression of A) markers of gut barrier function: *Cldn3* (one-way ANOVA $p<0.0001$) and *Ocln* (one-way ANOVA $p<0.05$) and, B) antimicrobial peptides. Mice were fed a control diet (n=6-7), a high-fat high-sucrose diet (HFHSD) (n=7-8), HFHSD with propyl propane thiosulfinate (PTS) at low dose (0.1 mg/kg day) (n=7-8) and high dose (1 mg/kg day) (n=7-8). Statistical analyses were performed by one-way analysis of variance (ANOVA) followed by *post hoc* Tukey's multiple comparison tests for normally distributed data, and Welch's correction was applied when variances were not equally distributed. Different superscript letters show statistical differences in the *post hoc* test when $p<0.05$.
